# 1543. Elevated HIV Risk/Low Risk Perception in Rural Colorado

**DOI:** 10.1093/ofid/ofad500.1378

**Published:** 2023-11-27

**Authors:** Amanda Rivera, Alexa Van Epern, Suzanne Fiorillo, Lisa Lawrence, Shynia Litzau, Lorna Allen, Katherine Frasca, Jose Castillo-Mancilla, Donna V McGregor

**Affiliations:** University of Colorado School of Public Health, Aurora, Colorado; University of Colorado Hospital, Aurora, Colorado; University of Colorado School of Medicine, Aurora, Colorado; University of Colorado School of Medicine, Aurora, Colorado; UCHealth, Westminster, Colorado; UCHealth University of Colorado Hospital, Aurora, Colorado; University of Colorado School of Medicine, Aurora, Colorado; University of Colorado-AMC; University of Colorado School of Medicine, Aurora, Colorado

## Abstract

**Background:**

The *increasing* impact of *HIV* in *rural populations* highlights the need for expanding HIV Pre-exposure Prophylaxis (PrEP) access in rural communities. Little is known about HIV risk perception, or PrEP awareness, and acceptability in rural populations.

**Methods:**

We analyzed baseline survey data from 69 residents as part of a larger study aiming to increase PrEP utilization in rural Colorado with virtual PrEP visits (TelePrEP) and mailed lab kits. The baseline survey on HIV risk, PrEP awareness, and access was conducted in 3 low-income rural Colorado counties from March 2022-February 2023. Residents were recruited from rural community settings, including a syringe service program, a substance treatment program, a migrant housing complex, a Native American Pow Wow, a college campus, and a public health clinic. All survey respondents were given the opportunity to enroll in TelePrEP services if eligible. Respondent demographics, HIV risk factors, risk perception, PrEP awareness, and access were examined. Descriptive statistics were used to characterize the cohort.

**Results:**

Seventy-five % of the rural cohort identified as female; 23.2% as male; and 1.5% as other gender. Thirty-three % of the cohort were White; 34.8% were Hispanic; 26.1% were Native American; and 2.9% were Black. Seventy-eight % self-identified as heterosexual; 13% as bisexual; 8.7% as homosexual. Sixty-seven % self–rated personal HIV risk as low, though almost a third of the cohort reported 2 or more sexual partners, 58% reported condomless sex, and 59% reported heavy alcohol or drug use, in the prior six months. Almost 40% of the cohort reported that they or a partner had a history of incarceration. Sixty-three % reported seeing a regular health care provider (HCP). Forty-two % had heard of PrEP previously, but only 5 respondents had heard of PrEP from an HCP. Seventy-two % of the cohort felt that taking PrEP was a good way to protect themselves from HIV; however, none of the respondents chose to enroll in PrEP services.

**Conclusion:**

Despite elevated risk for HIV acquisition and the majority having regular access to a health care provider, PrEP awareness and utilization was low in this cohort of rural residents. A gap in perceived versus objective HIV risk exists.

**Disclosures:**

**Lorna Allen, FNP-C**, MERCK: Grant/Research Support **Donna V. McGregor, MSN, ANP-BC**, Gilead Sciences: Grant/Research Support

